# Comparison of marker gene expression in chondrocytes from patients receiving autologous chondrocyte transplantation versus osteoarthritis patients

**DOI:** 10.1186/ar2218

**Published:** 2007-06-27

**Authors:** Reinout Stoop, Dirk Albrecht, Christoph Gaissmaier, Jürgen Fritz, Tino Felka, Maximilian Rudert, Wilhelm K Aicher

**Affiliations:** 1 NMI Natural and Medical Sciences Institute at the University of Tübingen, Markwiesenstraße, 72770 Reutlingen, Germany; 2 BG Center for Traumatology, Schnarrenbergstraße, 72076 Tübingen, Germany; 3 Center for Medical Research, Department of Orthopaedic Surgery, University of Tübingen, Waldhörnlestraße, 72072 Tübingen, Germany; 4 Department of Orthopaedic Surgery, Technische Universität München, Ismaninger Str., 81675 Munich, Germany

## Abstract

Currently, autologous chondrocyte transplantation (ACT) is used to treat traumatic cartilage damage or osteochondrosis dissecans, but not degenerative arthritis. Since substantial refinements in the isolation, expansion and transplantation of chondrocytes have been made in recent years, the treatment of early stage osteoarthritic lesions using ACT might now be feasible. In this study, we determined the gene expression patterns of osteoarthritic (OA) chondrocytes *ex vivo *after primary culture and subculture and compared these with healthy chondrocytes *ex vivo *and with articular chondrocytes expanded for treatment of patients by ACT. Gene expression profiles were determined using quantitative RT-PCR for type I, II and X collagen, aggrecan, IL-1β and activin-like kinase-1. Furthermore, we tested the capability of osteoarthritic chondrocytes to generate hyaline-like cartilage by implanting chondrocyte-seeded collagen scaffolds into immunodeficient (SCID) mice. OA chondrocytes *ex vivo *showed highly elevated levels of IL-1β mRNA, but type I and II collagen levels were comparable to those of healthy chondrocytes. After primary culture, IL-1β levels decreased to baseline levels, while the type II and type I collagen mRNA levels matched those found in chondrocytes used for ACT. OA chondrocytes generated type II collagen and proteoglycan-rich cartilage transplants in SCID mice. We conclude that after expansion under suitable conditions, the cartilage of OA patients contains cells that are not significantly different from those from healthy donors prepared for ACT. OA chondrocytes are also capable of producing a cartilage-like tissue in the *in vivo *SCID mouse model. Thus, such chondrocytes seem to fulfil the prerequisites for use in ACT treatment.

## Introduction

Hyaline articular cartilage is a tissue designed for weight bearing, shock absorption and providing the gliding surfaces needed for movement of joints. Since the self-renewal and repair capabilities of cartilage are very limited [1], even small injuries to articular cartilage can cause degeneration that eventually requires surgical management at later stages of cartilage destruction [2]. Current surgical treatments include tissue response techniques (for example, Pridie drilling, microfracturing), osteochondral transplantation and ultimately the implantation of artificial joints.

An additional treatment, the autologous chondrocyte transplantation (ACT) technique, was introduced more than a decade ago [3,4]. This technique is based on the isolation of chondrocytes from a small piece of knee cartilage taken from a non-load-bearing area, followed by *in vitro *expansion of these cells and their re-implantation into the defect area [5]. Guidelines of medical societies based on clinical experience suggest that larger defects (≥4 cm^2^) should be treated using the ACT method [6]. Since patients diagnosed with degenerative arthritis generally have larger cartilage defects in the patellofemoral contact area, ACT would be the preferred treatment for the regeneration of such large defects. While current International Cartilage Repair Society (ICRS) criteria do not recommend ACT as a therapeutic option for elderly patients or patients suffering from degenerative, reactive or inflammatory arthritis, a recent study using ACT to treat patients suffering from early degenerative arthritis indicates that this method might indeed be a therapeutic option for osteoarthritic lesions [7]. One major question remaining is whether osteoarthritic chondrocytes are changed irreversibly or, upon expansion under optimized conditions, are comparable with those cells that are currently used for ACT and are able to generate hyaline cartilage.

Molecular strategies for monitoring the gene expression patterns of chondrocytes destined for ACT were developed recently for the quality management of therapeutic cell culture and the safety of ACT patients [8,9]. To evaluate whether fundamental differences exist between osteoarthritic chondrocytes and cells currently used for the ACT procedure, we employed these quality management regimens and compared the expression of key factors for cartilage regeneration, including type I and II collagens, aggrecan, IL-1β and activin-like kinase (ALK)-1. We compared chondrocytes from osteoarthritis (OA) patients to chondrocytes from healthy donors directly after cell harvest (*ex vivo*) and to those from patients undergoing ACT after primary *in vitro *expansion (P0 cells) and first subculture (P1 cells). ALK-1 is a receptor involved in TGF-β signalling [10] and is proposed to be a marker for irreversible chondrocyte dedifferentiation [11]. The OA chondrocytes were prepared and expanded under the same good manufacturing practice protocols applied for ACT, except that autologous serum was not available from OA patients due to the regulations imposed by the local ethics committee. Therefore, clinical grade human AB serum was used instead of autologous serum for the *in vitro *culture of chondrocytes.

To determine whether OA chondrocytes were still capable of *in vivo *cartilage formation, we implantated collagen scaffolds seeded with these chondrocytes ectopically into severe combined immune deficient (SCID) mice. The formation of type II collagen and proteoglycan-rich hyaline cartilage-like tissue could be shown using histochemistry and immunohistochemical staining of implant sections.

We report that OA chondrocytes generated a proteoglycan and type II collagen-rich cartilaginous tissue when seeded onto a collagen scaffold at higher densities. We conclude that OA chondrocytes might be able to regenerate cartilage when applied under suitable conditions.

## Materials and methods

### Donors

Chondrocytes from OA patients were obtained from macroscopically intact cartilage areas of 29 patients undergoing knee joint implant surgery. Samples were taken from the intercondylar femoral notch (fossa intercondylica). The average age of the OA patients at the time of surgery was 67.2 ± 10.1 years (minimum 46 years, maximum 89 years). To compare the status of these cells with cells that are actually used for ACT, chondrocytes obtained from human articular cartilage biopsies from the femoral notch of 41 patients undergoing ACT were included in this study. All procedures followed the guidelines for ACT to treat chondral defects [6]. ACT surgery was performed as described previously [3]. The average age of the patients at the time of ACT was 32.3 ± 10.0 years (minimum 16 years, maximum 57 years).

Since all the chondrocytes from the ACT patients had to be used for expansion and transplantation, no ACT cells were available for analysis *ex vivo*. As a surrogate for ACT *ex vivo *controls, chondrocytes were obtained from the cartilage of six knee joints of individuals without any osteoarthritic symptoms (36.6 ± 12.5 years, minimum 23 years, maximum 50 years) either post mortem (*n *= 1) or after amputation (*n *= 5). The study was approved by the local ethics committee.

### Chondrocyte isolation and *in vitro *expansion

Cartilage samples, excluding the mineralized cartilage and the subchondral bone, were washed twice in PBS (BioWhittaker, Verviers, Belgium) and then minced. Extracellular matrix was enzymatically degraded overnight by incubation in DMEM/Ham's F12 medium (BioWhittaker; Verviers, Belgium) containing 2.5 mg/ml type II collagenase (Roche, Mannheim, Germany) and 5% serum at 37°C. Cell culture medium for ACT chondrocytes was supplemented with autologous serum, culture medium for OA chondrocytes with human AB serum. Isolated chondrocytes were resuspended by pipetting up and down several times and then filtered through a 100 μm mesh to remove undigested cartilage fragments and extracellular matrix debris. After centrifugation, the cells were resuspended in DMEM/Ham's F12 cell culture medium supplemented with either 10% autologous or AB serum and plated in cell culture flasks (BD Falcon, Heidelberg, Germany) at an initial density of 1,500 cells/cm^2^. At this point, some of the cells were harvested to provide *ex vivo *cells.

Chondrocytes were cultured at 37°C in humidified atmosphere containing 5% CO_2_. The cells were harvested after 10 to 12 days of expansion by trypsin-EDTA (BioWhittaker) treatment. Cell yields and viability were monitored by trypan blue staining using a Neubauer hematocytometer. At this time, cells were removed to determine gene expression patterns after primary expansion (P0), used for *in vivo *experiments, or cultured for an additional 12 to 14 days to provide first subculture (P1) cells. All procedures were performed according to the good manufacturing practice guidelines required for tissue engineering.

### Gene expression analysis

RNA was extracted and isolated from chondrocytes using the RNeasy mini kit according to the manufacturer's instructions (Qiagen Inc., Valencia, CA, USA). To isolate RNA from the cell-seeded scaffolds that were implanted into SCID mice, the scaffolds were frozen in 350 μl RLT buffer (Qiagen RNeasy Mini kit) supplemented with 10 μl/ml β-mercaptoethanol. Scaffolds were then homogenized using a micropestle (Eppendorf, Hamburg, Germany) and samples were frozen at -80°C until further isolation.

Complementary DNA (cDNA) was obtained by reverse transcription of 1 μg total RNA using oligo-dT primers and MuMLV reverse transcriptase (BD Clontech, Heidelberg, Germany). Reverse transcription was performed in a total volume of 20 μl at 42°C for 1 h in a thermocycler (Whatman Biometra, Göttingen, Germany). Expression of mRNA/cDNA levels was determined by quantitative real-time RT-PCR (qRT-PCR; LightCycler^®^, Roche) using specific target primers (Table 1) and FastStart DNA SybrGreen reagents (Roche) according to the protocols provided. The amplification of cDNA was performed in 35 PCR cycles: after 5 initial cycles (95°C 10 s, 68°C 10 s, 72°C 16 s, temperature transition rate 20°C/s) the annealing temperature was dropped in consecutive cycles to 60°C with a step size of 0.5°C.

**Table 1:** PCR primer sequences

Gene product	Sequences	Accession number	Position	Product (base-pairs)
Collagen I(a2)	Up: 5'-CTGGTCCTTCTGGTCCTGTTG	NM_000089	3,413	
	Low: 5'-GTGCGAGCTGGGTTCTTTCTA		3,957	544
				
Collagen II(a1)	Up: 5'-CTGGCTCCCAACACTGCCAACGTC	NM_033150	4,070	
	Low: 5'-TCCTTTGGGTTTGCAACGGATTGT		4,483	413
				
Collagen X	Up: 5'-ACCCAAGAGGTGCCCCTGGAATAC	NM_000493.2	1,416	
	Low: 5'-CCTGAGAAAGAGGAGTGGACATAC		2,117	701
				
Aggrecan	Up:5'-AGCTGGGTTCGGGGCATCT	NM_013227	6,039	
	Low:5'-TGGTAGTCTTGGGCATTGTTGTTGA		6,839	800
				
IL-1β	Up:5'-ATGGCAGAAGTACCTAAGCTCGC	NM_000576	87	
	Low:5'-ACACAAATTGCATGGTGAAGTCAGTT		889	802
				
ALK-1	Up: 5'-CGGCTCCCTCTACGACTTTCT	Z_22533	1,128	
	Low: 5'-CAGCACTCCCGCATCATCT		1,479	570
				
GAPDH	Up: 5'-TGAAGGTCGGAGTCAACGGATTTGGT	NM_002046	113	
	Low: 5'-CATGTGGGCCATGAGGTCCACCAC		1,095	983

ALK = activin-like kinase; GAPDH = glyceraldehyde-3-phosphate dehydrogenase.

To monitor the specificity of the amplification, melting curve analysis was performed after each PCR. In addition, some samples were separated by electrophoresis and visualized on agarose gels to confirm the size and purity of the PCR products. Amplification of glyceraldehyde-3-phosphate dehydrogenase (GAPDH) encoding cDNA and serial dilutions of a recombinant standard with a known DNA concentration (Roche) served as controls in each PCR. Data show the mean of the mRNA expression levels of the gene investigated normalized by the respective GAPDH signal and recombinant standard in each individual sample and PCR reaction. To show the relative copy numbers of the different genes investigated (ranging from more than 10^6 ^to less than 1 copy/μl cDNA) qRT-PCR data are shown on a log scale. This required an adjustment of all normalized values by a factor of 100,000. Statistical evaluation of the data was performed by a Mann-Whitney U test. Groups were considered statistically different when the probability values *p *were equal to or smaller than 0.05.

### *In vivo *cartilage formation

To investigate the capability of OA chondrocytes to form cartilage under *in vivo *conditions, primary culture cells (P0 cells) from three osteoarthritic donors (OA donor 1, age 78 years; OA donor 2, age 68 years; OA donor 3, 50 years) and two healthy donors (healthy donor 1, age 50 years; healthy donor 2, age 42 years) were harvested by mild enzymatic detachment, washed, counted, resuspended in cell culture medium and seeded onto a biphasic collagen matrix (Jotec AG, Hechingen, Germany). The scaffold consisted of a bovine collagen membrane on one side and a porous collagen sponge on the other side. The sponge side of the scaffold was seeded with 1 × 10^6 ^or 3 × 10^6 ^OA chondrocytes/cm^2 ^or 1 × 10^6 ^healthy chondrocytes/cm^2^.

The cell-scaffold constructs were then cultured *in vitro *for an additional 4 days, after which they were implanted subcutaneously into female CB-17/Lcr SCID mice aged 10 to 12 weeks (Charles-River Wiga, Sulzfeld, Germany; *n *= 3 per group). The mice were anesthetized using ketamine and xylazine (1 ml 10% ketamine (WDT eG, Garbsen, Germany) and 1 ml xylazine (Rompun^®^, WDT eG) in sterile PBS; 0.1 ml/10 g body weight subcutaneously). Two scaffolds were implanted subcutaneously at the back of each mouse through a small incision in the neck region. Empty scaffolds were used as controls. The mice were kept in isolator cages in an air-conditioned specific pathogen free facility on an unrestricted diet. After 8 weeks the mice were sacrificed using CO_2_, and the constructs were harvested and fixed in 10% formalin buffered with 0.1 M phosphate buffer (pH 7.4). All procedures were approved by the local animal care committee.

In an additional experiment, scaffolds seeded with cells from four OA patients or three ACT patients were implanted into the mice and harvested after eight weeks for mRNA isolation.

### Histological analysis

After fixation, the constructs were embedded in Tissue Tec compound (Sakura, Zoeterwoude, The Netherlands) and 7 μm sections were cut with a cryomicrotome (Jung/Leica Instruments, Nussloch, Germany). To determine if synthesis of cartilage-like tissue had occurred, we stained sections with safranin O/fast green to show the presence of proteoglycans. Type I and type II collagen was also visualized using standard immunohistochemistry. Type I collagen was detected using the 1-855 monoclonal antibody (IgG2a, ICN Pharmaceuticals, Aurora, OH, USA), type II collagen using the II-II6B3 monoclonal antibody (IgG1, kappa light chain) [12] followed by a biotin-labeled horse anti-mouse serum (Vector, Burlingame, CA, USA). A biotin-streptavidin detection system (Vectastain Elite Kit, Vector) was used according to the manufacturer's recommendations. The II-II6B3 antibody was obtained from the Developmental Studies Hybridoma Bank maintained by the Department of Pharmacology and Molecular Sciences, Johns Hopkins University School of Medicine, Baltimore, MD, and the Department of Biological Sciences, University of Iowa, Iowa City, IA, under contract NO1-HD-2-3144 from the NICHD.

Hypertrophic chondrocytes were detected by staining for alkaline phosphatase (ALP). Sections were washed in ALP buffer (0.1 M Tris-HCl, 0.1 M NaCl, 5 mM MgCl_2_, pH 9.0) and then incubated with 3.5 μl 5-bromo-4-chloro-3-indolyl phosphate (50 mg/ml, Sigma Aldrich, Taufkirchen, Germany) and 4.5 μl nitroblue tetrazolium (50 mg/ml, Sigma-Aldrich) per ml ALP buffer. The reaction was stopped by washing with PBS.

## Results

### Analysis of gene expression patterns in chondrocytes *ex vivo*

**Figure 1:**
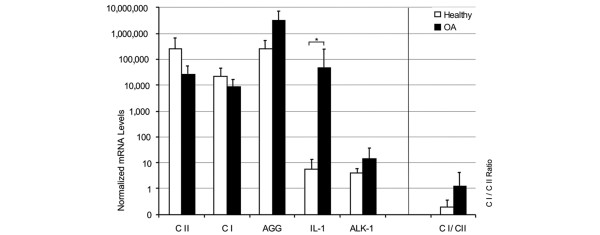
Gene expression patterns of chondrocytes *ex vivo*. Chondrocytes were isolated from cartilage of healthy individuals (*n *= 6, white bars) or osteoarthritis (OA) patients (*n *= 20, black bars). The *ex vivo *gene expression of type I and II collagen (CI and CII, respectively), aggrecan (AGG), IL-1β and activin-like kinase (ALK)-1 was determined using qRT-PCR. The mRNA levels were normalized to GAPDH and amplified by a factor of 10^6^. The collagen type I to collagen type II mRNA ratio was calculated as a measure for the differentiation status of the chondrocytes. Statistically significant differences (*p *< 0.05) are marked by asterisks (*).

In the first set of measurements, gene expression patterns were investigated in cells directly after isolation from cartilage. As all of the chondrocytes obtained from the ACT patients were needed for expansion and subsequent transplantation, *ex vivo *ACT chondrocytes were not available for experimental investigation. Instead, cells from healthy cartilage served as surrogates. *Ex vivo *chondrocytes from healthy donors showed a prominent type II collagen signal by qRT-PCR and a somewhat weaker mRNA expression than OA chondrocytes (Figure 1). Type I collagen encoding mRNA was expressed to a lesser extent, resulting in very low type I to type II collagen ratios in both groups. The expression of aggrecan mRNA was high in both chondrocyte populations *ex vivo*. In *ex vivo *OA chondrocytes it exceeded the mRNA amounts found in chondrocytes from healthy donors (Figure 1), indicating that cells from both groups were in a highly differentiated state. This was confirmed by the low expression of ALK-1, a marker for chondrocyte dedifferentiation, in both groups (Figure 1). However, despite having similar collagen and aggrecan expression patterns, significant differences in IL-1β mRNA levels could be seen between healthy and OA cells: in OA chondrocytes, *ex vivo *IL-1β levels were more than 8,000 times (*p *< 0.05) higher than in the healthy controls (Figure 1).

### Analysis of gene expression patterns in OA and ACT chondrocytes after primary culture

**Figure 2:**
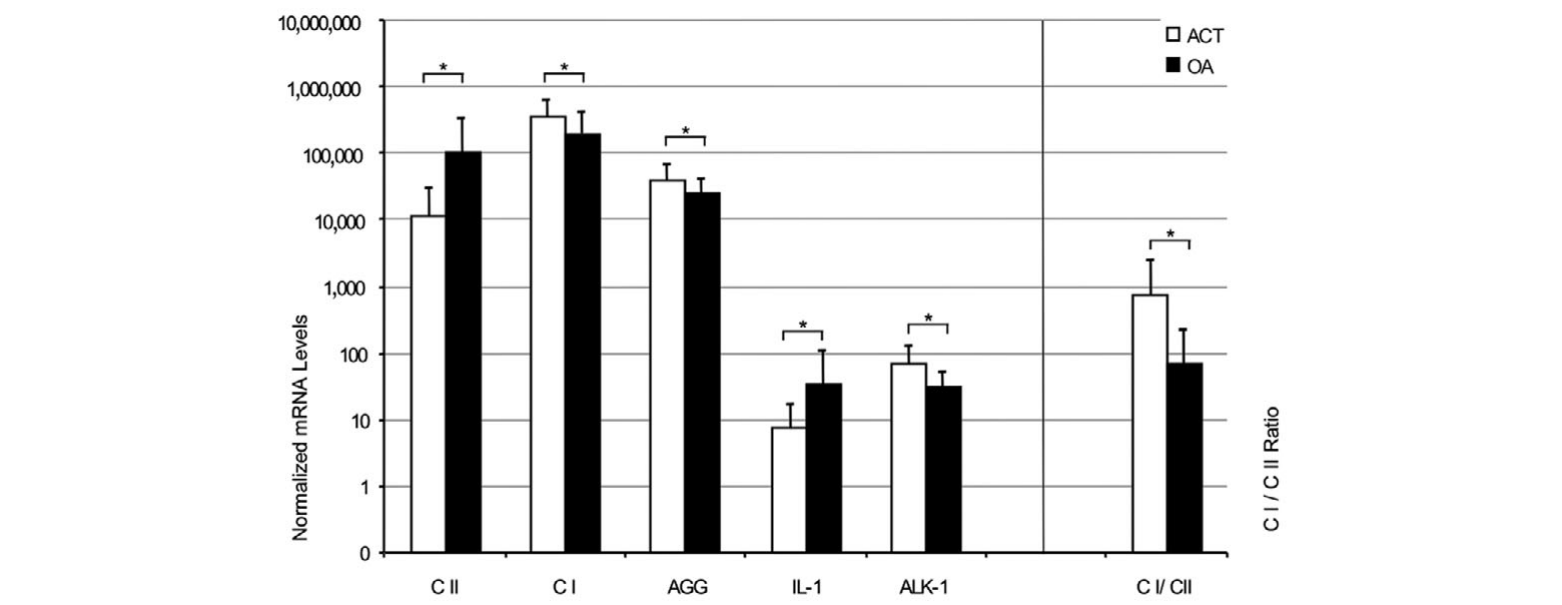
Gene expression pattern of chondrocytes after primary culture (P0). Chondrocytes isolated from cartilage of patients undergoing autologous chondrocyte transplantation (ACT; *n *= 40, white bars) or osteoarthritis (OA) patients (*n *= 26; black bars) were expanded in primary culture for 10 to 12 days and the gene expression of type I and II collagen (CI and CII, respectively), aggrecan (AGG), IL-1β and activin-like kinase (ALK)-1 was determined using qRT-PCR. The mRNA levels were normalized to GAPDH and amplified by a factor of 10^6^. In comparison to cells *ex vivo*, the collagen type I to collagen type II mRNA ratio is increased, especially in ACT chondrocytes. Statistically significant differences (*p *< 0.05) are marked by asterisks (*).

Interestingly, the high IL-1β expression observed *ex vivo *in OA chondrocytes dropped strongly (1,448-fold) after 10 to 12 days of *in vitro *culture to expression levels only slightly higher than the IL-1β expression of healthy chondrocytes *ex vivo *or of ACT chondrocytes after primary culture. Although the increase in type I collagen and the decrease in aggrecan expression levels during culture suggested a slight dedifferentiation of the OA chondrocytes, culture of the OA chondrocytes also resulted in a four-fold increase in type II collagen expression compared to the *ex vivo *values (Figure 2). These levels were almost nine times higher than those in ACT cells cultured using the same protocol. Since the type I collagen expression levels differed only slightly between OA and ACT chondrocytes (Figure 2), this led to a significantly better type I/type II collagen ratio in OA chondrocytes than in the OA cells. Combined with the slightly lower ALK-1 expression in the OA cells, this suggests that the phenotypic state of OA chondrocytes is at least comparable to, if not better than, that of ACT cells at the stage where the latter are used for transplantation back into the patient.

### Gene expression in OA chondrocytes after *in vitro *expansion

**Figure 3:**
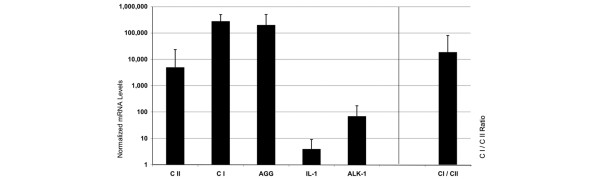
Gene expression pattern of chondrocytes after first passage (P1). Chondrocytes from cartilage of osteoarthritis (OA) patients (*n *= 18, black bars) were subcultured in a first passage and further expanded until they reached confluence after an additional 12 to 14 days. The gene expression patterns were enumerated by qRT-PCR for type I and II collagen (CI and CII, respectively), aggrecan (AGG), IL-1β and activin-like kinase (ALK)-1 as indicated. The mRNA levels were normalized to GAPDH and amplified by a factor of 10^6^. The ratio of type I to type II collagen mRNA levels continue to increase in P1 OA chondrocytes.

Since defect sizes in OA cartilage are expected to be larger than those currently treated using the ACT method, the number of cells required for ATC will be greater as well. Therefore, we further expanded the OA chondrocytes and analyzed the gene expression patterns in first subculture cells (P1). We found that the expression of type II collagen mRNA was much weaker (23-fold; Figure 3) in P1 OA chondrocytes than in P0 cells. Although the expression of type I collagen remained constant, this still led to a 270-fold increase of the type I to type II ratio in P1 OA cells compared to the P0 OA cells, an indicator for dedifferentiation of the cells. It is interesting to note that in P1 OA chondrocytes, IL-1β expression decreased further to levels found in healthy chondrocytes *ex vivo *and in primary culture ACT chondrocytes (Figure 3).

### *In vivo *cartilage formation

**Figure 4:**
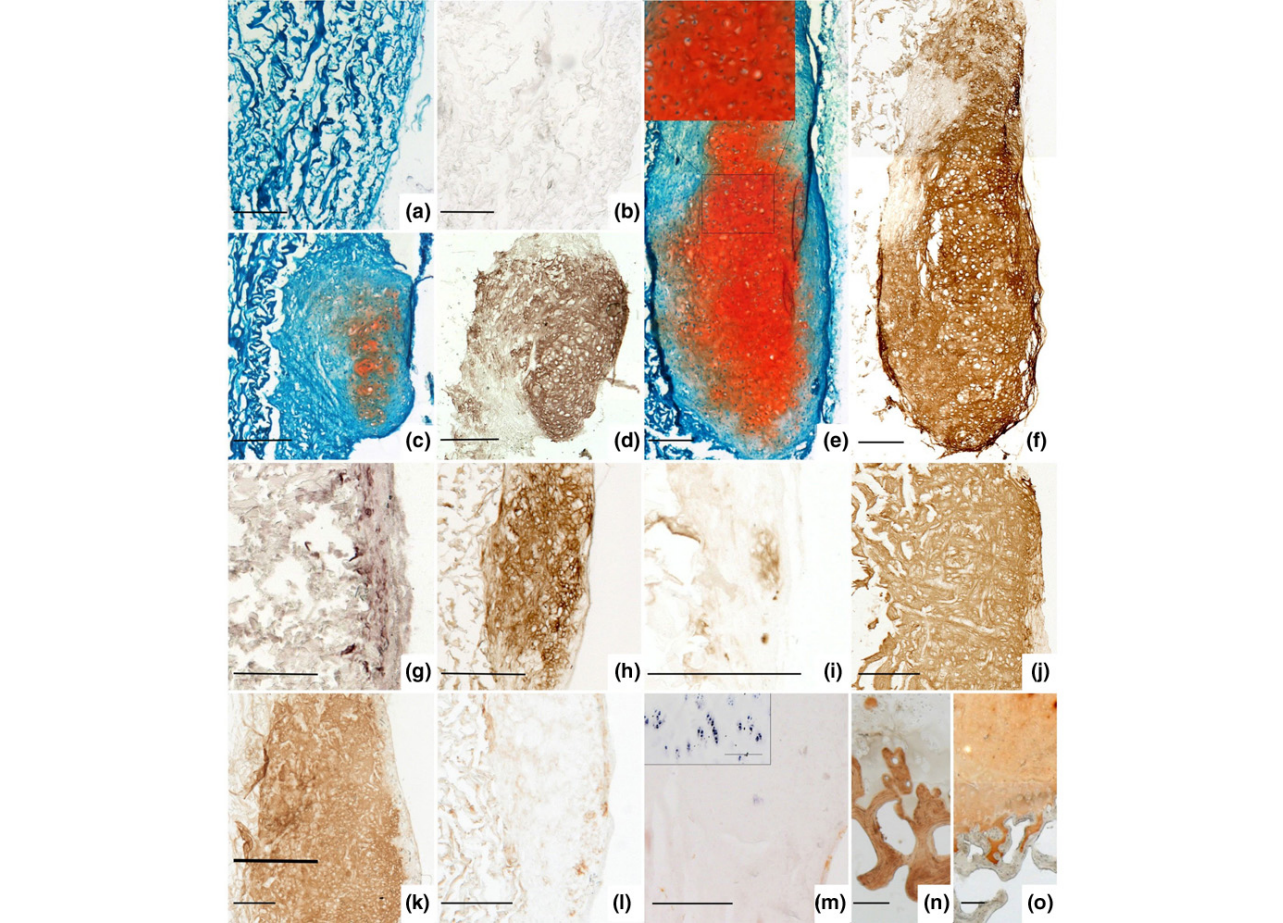
*In vivo *cartilage formation of osteoarthritic chondrocytes seeded on collagen scaffolds. Collagen scaffolds were seeded with human chondrocytes, implanted subcutaneously into SCID mice and harvested after eight weeks. **(a,b) **On empty scaffolds no cartilage formation occurred, as shown by the absence of dense Safranin O (a) or type II collagen (b) staining. **(c,d) **On scaffolds seeded with 1 × 10^6 ^osteoarthritic chondrocytes from osteoarthritis (OA) donor 1, moderate amounts of cartilage-like proteoglycan (c) and type II collagen (d) containing tissue could be detected. **(g,i) **However, hardly any type II collagen positive tissue was formed in scaffolds seeded with 1 × 10^6 ^chondrocytes from OA donors 2 (g) and 3 (i). **(d,f,h,j) **Seeding scaffolds at the higher density of 3 × 10^6 ^chondrocytes/cm^2 ^resulted in the formation of type II collagen- and proteoglycan-rich cartilage by cells from all three donors in amounts comparable to those produced by **(k) **1 × 10^6 ^healthy chondrocytes. **(l,m) **No type I collagen (l) or alkaline phosphatase activitiy (m) could be detected in these tissues. (c-f) OA donor 1, 78 years. (g,h,l) OA donor 2, 68 years. (i,j,m) OA donor 3, 50 years. (k) Healthy donor, 40 years. (a,c,e) Safranin O staining. (b,d,e-k) Type II collagen immunostaining. (m-o) Positive controls (OA cartilage, cartilage-bone interface) for type I (n), type II (o) and alkaline phosphatase activity (insert in (m)). Bar = 250 μm.

To investigate whether P0 OA chondrocytes might be employed for the regeneration of cartilage defects by ACT, we seeded such cells onto biphasic collagen scaffolds and implanted them subcutaneously into immune deficient mice. This scaffold consists of a very slowly degrading membrane and a porous region (sponge) that is normally replaced by cartilage-like tissue within eight weeks. After eight weeks of implantation, no cartilage formation could be observed in the empty control scaffolds (Figure 4a,b). Some scattered cells of murine origin were present inside the scaffold (data not shown). In scaffolds seeded with the lowest tested density of chondrocytes (1 × 10^6 ^cells/cm^2^), cartilage-like tissue containing proteoglycans (Figure 4c) and type II collagen (Figure 4d) was formed only in scaffolds seeded with cells from OA donor 1. In scaffolds seeded with cells from the other two OA donors (Figure 4g,i) only isolated cells staining positive for type II collagen could be observed, and there was insufficient cartilage formation. However, when chondrocytes were seeded at a higher density (3 × 10^6 ^cells/cm^2^), cartilage was generated by cells from all three donors (Figure 4e,f,h,j), in levels similar to those found in the scaffolds seeded with 1 × 10^6 ^healthy chondrocytes/cm^2 ^(Figure 4k; one sample of three healthy donors). In these samples the spongy part of the scaffold was completely replaced by hyaline-like cartilage, as indicated by the presence of cells with a round, chondrocyte-like morphology embedded in proteoglycan – (Figure 4e) and type II collagen-rich tissue (Figure 4f,h,j). Hardly any type I collagen could be detected in the newly formed cartilage (Figure 4l). The absence of cell clustering (insert in Figure 4e), ALP activity (Figure 4m) and of large, hypertrophic chondrocytes (insert in Figure 4e) suggest that these cells show no inclination to become hypertrophic or to retain OA characteristics.

### Gene expression of chondrocytes in scaffolds

**Figure 5:**
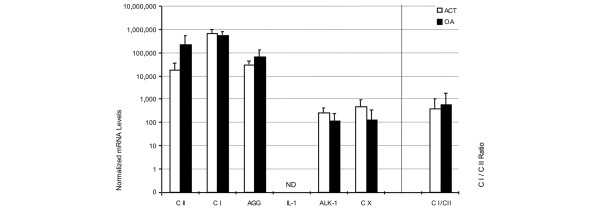
Gene expression pattern of chondrocytes after *in vivo *inoculation in scaffolds. Chondrocytes from healthy donors (*n *= 3, white bars) and from osteoarthritis (OA) patients (*n *= 4, black bars) were expanded in primary culture, seeded onto scaffolds and incubated for four days *in vitro*, followed by implantation for eight weeks subcutaneously in SCID mice. Scaffolds were harvested and RNA was extracted from the cells to investigate the gene expression patterns by qRT-PCR for type I and II collagen (CI and CII, respectively), aggrecan (AGG), IL-1β and activin-like kinase (ALK)-1 as indicated. The mRNA levels were normalized to GAPDH and amplified by a factor of 10^6^. Cells from healthy donors expressed slightly more collagen, but no significant differences in gene expressions or differences in the type I to type II collagen ratio were observed between the ACT versus OA cells. ND, not detectable.

To investigate the regenerative potential in OA chondrocytes in comparison to ACT cells in more detail, cells from both cohorts were expanded in primary culture, seeded onto collagen scaffolds, and implanted subcutaneously into SCID mice as described above. After eight weeks in situ, the scaffolds were harvested to determine the gene expression patterns by qRT-PCR. Samples derived from ACT patients showed no significant differences in mRNA expression levels of any of the genes investigated compared to cells from OA patients (Figure 5). Interestingly, the expression of IL-1β mRNA remained below qRT-PCR detection levels in all the samples. Furthermore, only low levels of type X collagen mRNA expression could be detected, suggesting that the implanted cells did not become hypertrophic. We conclude from these data that chondrocytes harvested from intact sites of articular cartilage of OA patients are not significantly different with respect to the factors investigated in this study and seem to retain at least some regenerative potential.

## Discussion

The advent of reliable cell culture techniques raised hopes that tissue engineering might be able to cure any type of cartilage damage, regardless of pathology, degeneration of tissue, health status and age of patient [13]. In a recent study, regeneration of cartilaginous tissue was reported using chondrocytes from elderly donors [14] and ACT can be successful in certain patients suffering from early stage arthritis [7]. This encouraged us to investigate whether OA chondrocytes might possibly be used for ACT.

Our data suggest that OA chondrocytes *ex vivo *are in a differentiation state similar to that of healthy chondrocytes, as shown by their low expression levels of ALK-1, similar expression levels of type I collagen, and high expression of type II collagens and aggrecan mRNA. But OA chondrocytes *ex vivo *contain significantly more IL-1β encoding mRNA. This could cause major problems for their use in the ACT procedure, since IL-1β is known to induce chondrocyte-mediated cartilage degradation [15,16] and to reduce type II collagen expression [17]. Factors activating IL-1β expression *in vivo *– as reflected by the highly elevated IL-1β mRNA amounts found *ex vivo *– may contribute not only to the degradation of articular cartilage during OA but at the same time induce a catabolic situation in a transplant after ACT. Therefore, treatment of the osteoarthritic joint prior to and directly after ACT by blocking inflammatory processes or inhibiting catabolic factors should be taken into consideration.

Upon primary culture of the OA chondrocytes, a strong reduction of IL-1β expression was accompanied by an increase in type II collagen expression, even exceeding the levels found in ACT chondrocytes cultured under the same conditions. It is unclear whether this increase in type II collagen expression results from a loss of an inhibitory effect of IL-1β or if this reflects a general activation of gene expression described in OA chondrocytes *in vivo *and *ex vivo *[18]. In addition to an increase in type II collagen expression upon culture of the OA cells, we also observed an increase in type I collagen and ALK-1. In an earlier study [19], chondrocytes from OA patients in first or second passage cultures did not differ significantly from chondrocytes obtained from healthy donors with respect to their type II and type I collagen expression patterns. In first passage cells, the expression of transcription factors regulating collagen, including SOX-5, -6, and -9, appeared to be even higher in OA chondrocytes. These findings are consistent with our data, as a significantly lower ALK-1 expression and collagen ratio were observed. However, an upregulation of type X collagen expression has been reported in OA cells in comparison with healthy chondrocytes [19]. This suggests that OA chondrocytes in primary culture show fewer signs of dedifferentiation but rather move towards a more hypertrophic phenotype, which might limit their use for tissue engineering. However, in our *in vivo *experiments, the cells did not show any ALP activity, which is another marker for hypertrophic chondrocytes [20]. This finding argues against a progression of the cells towards a stage of hypertrophy. Further differences in the expression of type X collagen between cells from ACT and OA patients were not observed. Our results also show that the production of high-quality ACT cells from osteoarthritic cartilage does not necessarily require additional manipulation of the cells such as alginate or agarose culture to stabilize the chondrogenic phenotype in these cells [21,22].

The downregulation of IL-1β expression in OA chondrocytes upon expansion implies that these cells are not irreversibly changed. It is possible that the osteoarthritic tissue induced the elevated IL-1β expression observed *ex vivo*. For example, chondrocytes are known to upregulate the production of their own pro-inflammatory cytokines, including IL-1β and tumor necrosis factor-α, under the influence of proinflammatory cytokines present in the synovial tissues of patients with early OA [23,24], mechanical stress [25], and breakdown products from the cartilage matrix [26,27]. At the same time, their responsiveness to IL-1β is reduced [28], making these cells less sensitive to autocrine IL-1β during *in vitro *expansion. This may contribute to a normalization of IL-1β expression *in vitro *as well. Interestingly, in cells seeded onto the type I collagen scaffold, IL-1β mRNA was basically below detection levels eight weeks after implantation. Therefore, to ensure the success of ACT in OA joints, the control of articular environment will be of the utmost importance. Control of inflammatory stimuli in the affected joint and the removal of any degenerated cartilage surrounding the primary defect probably will be as important as the expansion of high quality autologous cells.

Using the SCID mouse model, we were able to show that OA chondrocytes seeded onto collagen scaffolds were capable of producing a hyaline cartilage *in vivo*. However, higher seeding densities (3 × 10^6 ^cells/cm^2^) were needed than those currently used for ACT procedures (1 × 10^6 ^cells/cm^2^). Similar results were found by Tallheden and colleagues [29] using a scaffold based on hyaluronic acid. Although the reason for this phenomenon is unclear, the higher inoculation density might compensate for reduced mitotic activity, lower metabolic activity or elevated cell death of OA cells in the scaffolds [30,31].

However, it seems that patient age by itself is not a major factor for tissue formation using OA chondrocytes. The chondrocytes from one patient included in this study (78 years of age) showed better *in vivo *tissue formation at lower inoculation density than those of younger donors (68 and 50 years of age). However, the influence of donor age on cartilage regeneration must be addressed in more detail in future studies.

Although our data suggest that chondrocytes from macroscopically intact cartilage of OA patients are of sufficient quality themselves, a number of additional problems will need to be solved before the ACT technique can become a viable treatment option for osteoarthritic cartilage. For example, OA defects are likely to be larger in size than most lesions currently treated with ACT. This means that greater numbers of cells are needed for the repair of cartilage damage in OA. We confirmed that extended expansion of OA chondrocytes beyond the P0 stage was marked by a strong reduction in type II collagen expression, upregulation of type I collagen expression and a slightly higher expression of ALK-1, showing ongoing dedifferentiation *in vitro*. Since redifferentiation of dedifferentiated, ALK-1^high ^chondrocytes resulted in a fibrous repair tissue [11], passaged OA chondrocytes are more likely to regenerate a fibrous cartilage. Here, the harvest of additional donor cells from the respective joint might be a better way of increasing the number of cells available for expansion in order to cover the rather large defects seen in OA. However, the additional damage to the joint resulting from the larger number of biopsies will have to be balanced carefully against the benefits of such an operation.

Furthermore, the challenge of preparing enough donor cells from an osteoarthritic joint and additional problems, such as joint stability, bone changes, and synovial inflammation, will have to be addressed to optimize cartilage regeneration. One subgroup of OA patients in which these problems might be more solvable comprise patients with a unilateral, varus or valgus OA of the knee. In these patients, sufficient cartilage is available to be used as donor material, the joint environment is probably not as catabolic as in end-stage OA, and most importantly, it is possible to correct the cause of the OA by adjusting the joint axis through osteotomy. Therefore, this group of patients might benefit from treatment using the ACT method.

## Conclusion

Our data suggest that chondrocytes from macroscopically intact cartilage of OA patients can be expanded *in vitro *in a quality suitable for scaffold-augmented ACT, although higher initial cell densities were needed to ensure sufficient cartilage formation. However, controlling the environment within these joints will be of the utmost importance in ensuring the success of ACT in OA patients. Hopefully, the development of novel tissue engineering strategies, such as the anti-inflammatory or growth-factor-releasing scaffolds currently under investigation by numerous groups, will ensure the right environment to allow the transplanted chondrocytes to restore the cartilage defect.

## Abbreviations

ACT = autologous chondrocyte transplantation; ALK = activin-like kinase; ALP = alkaline phosphatase; DMEM = Dulbecco's modified Eagle's medium; GAPDH = glyceraldehyde-3-phosphate dehydrogenase; IL = interleukin; OA = osteoarthritis; PBS = phosphate buffered saline; qRT-PCR = quantitative real-time RT-PCR; SCID = severe combined immune deficient.

## Competing interests

TETEC AG is a tissue engineering company. CG and JF have stock holdings in TETEC and have received salaries from the company. RS and WKA have received research funding and royalties, respectively. The terms of the financial support from TETEC included freedom for authors to reach their own conclusions, and an absolute right to publish the results of their research, irrespective of any conclusions reached. The remaining authors declare that they have no competing interests.

## Authors' contributions

RS participated in the design of the study, carried out animal experiments, evaluated histology and drafted part of the manuscript. DA collected human cartilage samples and participated in the animal experiments. TF performed part of the qRT-PCR analyses. CG, JF and MR collected human cartilage samples and contributed to the interpretation and discussion of data. WKA conceived the study, performed the gene profiling and drafted part of the manuscript. All authors read and approved the manuscript.
